# Multi-GNSS Combined Orbit and Clock Solutions at iGMAS

**DOI:** 10.3390/s22020457

**Published:** 2022-01-08

**Authors:** Wei Zhou, Hongliang Cai, Guo Chen, Wenhai Jiao, Qianqian He, Yuguo Yang

**Affiliations:** 1 Beijing Institute of Tracking and Telecommunications Technology (BITTT), 26 Beiqing Road, Beijing 100094, China; zhouwei_0611@163.com (W.Z.); caibanyu@126.com (H.C.); jiaowh@beidou.gov.cn (W.J.); 2 GNSS Research Center, Wuhan University, 129 Luoyu Road, Wuhan 430079, China; 3 China Electronics Technology Group Corporation No.15th Research Institute, No.211 Beisihuan Middle Road, Beijing 100083, China; heqq.90629@163.com; 4 School of Space Science and Physics, Shandong University, Weihai 264209, China; yyguo514@163.com

**Keywords:** iGMAS, multi-GNSS, orbit combination, clock combination, SLR residual, precise point positioning

## Abstract

Global navigation services from the quad-constellation of GPS, GLONASS, BDS, and Galileo are now available. The international GNSS monitoring and assessment system (iGMAS) aims to evaluate the navigation performance of the current quad systems under a unified framework. In order to assess impact of orbit and clock errors on the positioning accuracy, the user range error (URE) is always taken as a metric by comparison with the precise products. Compared with the solutions from a single analysis center, the combined solutions derived from multiple analysis centers are characterized with robustness and reliability and preferred to be used as references to assess the performance of broadcast ephemerides. In this paper, the combination method of iGMAS orbit and clock products is described, and the performance of the combined solutions is evaluated by various means. There are different internal precisions of the combined orbit and clock for different constellations, which indicates that consistent weights should be assigned for individual constellations and analysis centers included in the combination. For BDS-3, Galileo, and GLONASS combined orbits of iGMAS, the root-mean-square error (RMSE) of 5 cm is achieved by satellite laser ranging (SLR) observations. Meanwhile, the SLR residuals are characterized with a linear pattern with respect to the position of the sun, which indicates that the solar radiation pressure (SRP) model adopted in precise orbit determination needs further improvement. The consistency between combined orbit and clock of quad-constellation is validated by precise point positioning (PPP), and the accuracies of simulated kinematic tests are 1.4, 1.2, and 2.9 cm for east, north, and up components, respectively.

## 1. Introduction

Over the past decade, apart from the ongoing modernization of GPS and GLONASS, two new global navigation satellite systems (GNSSs) have been developed, namely the BeiDou navigation satellite system (BDS-3) of China and the Galileo system of Europe. Both BDS-3 and Galileo have already been providing positioning, navigation, timing (PNT), and other services to global users. The ‘big 4’ GNSSs allow users to have more choices to access reliable and accurate PNT services from multi-GNSS [1,2,3]. Precise multi-GNSS orbit and clock products are available from the international GNSS service (IGS) [4,5,6,7,8]. Combining the products from different analysis centers (ACs) improves the stability and continuity compared with the individual AC products, which enables the use of GNSS constellations for scientific research, technical tests, and various applications related to satellite navigation. Furthermore, the combined products can be used as references to objectively evaluate the service performance of the multi-constellation.

The combined solutions of GNSS orbit and clocks were firstly proposed by IGS, due to its robustness compared with the solution from a single AC. Seven analysis centers have been contributing to IGS with GPS precise orbit and Earth rotation parameters (ERP) products since 1993. To ensure the consistency of orbit product and IGS reference, a set of ERPs were used to apply an orientation alignment correction to the GPS final orbit products from different ACs, and then the weighted average orbit was used as the official IGS product [9,10]. As an extension of the International Terrestrial Reference Frame (ITRF), the IGS combined orbit product provides a good way to define, maintain, and transmit coordinate datum. With IGS ACs providing station coordinate products, the orientation differences of orbit products among ACs can be corrected by coordinate rotation parameters. Since 2000, IGS has used the rotation parameters of the station coordinate products relative to the IGS frame to align the frame before the orbit combination [11,12,13].

For the combined solution of multi-GNSS orbits, two alignment methods of the orbits to ITRF using ERP and individual orbit/station coordinates, respectively, which initially obtained IGS combined multi-system orbit products [14,15]. However, in that study, all systems were treated as a whole for orbit combination, and the differences of product frame and orbit accuracy between different analysis centers or systems were ignored, maybe resulting in unreasonable weight distribution of ACs. In order to deal with multi-GNSS orbit and clock combination, the next version of the combination software of IGS analysis center coordinator also underwent an update [16]. The experimental multi-GNSS orbit combined solutions of MGEX are available at https://igs.org/acc/experimental-multi-gnss-combinations/ (accessed on 30 December 2021), and the orbits were assessed by Sośnica et al. using SLR data. The standard deviations of 2.3, 2.9, 8.7, 5.1, 4.0, and 7.2 cm are achievable for Galileo, GLONASS, BDS GEO, BDS IGSO, BDS MEO, and QZSS, respectively [17].

In terms of IGS clock combination, due to the correlations of the radial orbit component and the clock estimates, orbit errors are mapped to the clock parameters. It is essential that the compatibility corrections are applied to ACs’ clock solutions before clock combination to maintain orbit/clock consistency [18,19]. However, the combined strategy of current IGS GPS clocks only considers the overall linear systematic errors between analysis centers rather than the clock offset bias among satellites, which ultimately affects the weight determination based on posterior variance statistics [19,20]. With the rapid development of PPP ambiguity resolution (AR), the combination strategy of PPP-AR products including satellite clock and bias corrections from ACs are described by Banville et al. [21].

The robust precise orbit and clock products are usually used to assess the performance of GNSS broadcast ephemerides. In order to obtain assessment results for BDS, as well as other GNSS for comparison, robust precise combined orbit and clock solutions are conducted by iGMAS. The orbit accuracy of GPS and GLONASS is better than 2 cm (relative to IGS orbit). BDS-2 IGSO/MEO and Galileo satellites can achieve an orbit precision of better than 10 cm, whereas the accuracy of BDS-2 GEO orbit is about 1 m [22,23]. However, the characteristics of orbit error are not assessed by SLR data in the previous study.

The precise orbit and 30s-sampling clock products for BDS-2/BDS-3, GPS, GLONASS and Galileo have been provided by multiple iGMAS ACs since the middle of 2019. Moreover, some adjustments were applied by analysis centers of iGMAS. Consequently, a new comprehensive evaluation of the performance of the corresponding iGMAS combined orbit and clock products is required. This study focuses on the combination processing and quality evaluation of newest iGMAS multi-GNSS orbit and clock products. The structure of this paper is as follows. Section 2 presents an overview of iGMAS organization structure and the feature of product service. Then, the product combination method proposed in this study is introduced in Section 3. In Section 4, we generate the combined multi-GNSS orbit and clock products based on the products provided by multiple ACs, and present a detailed analysis of the accuracy of iGMAS combined products in terms of internal precision and comparison with IGS products. The combined orbit products are also assessed by Satellite Laser Ranging (SLR) observations. The consistency between the combined orbit and clock is assessed by precise point positioning in this section. Finally, conclusions and a summary are provided in Section 5.

## 2. Overview of iGMAS ISC

**Figure 1:**
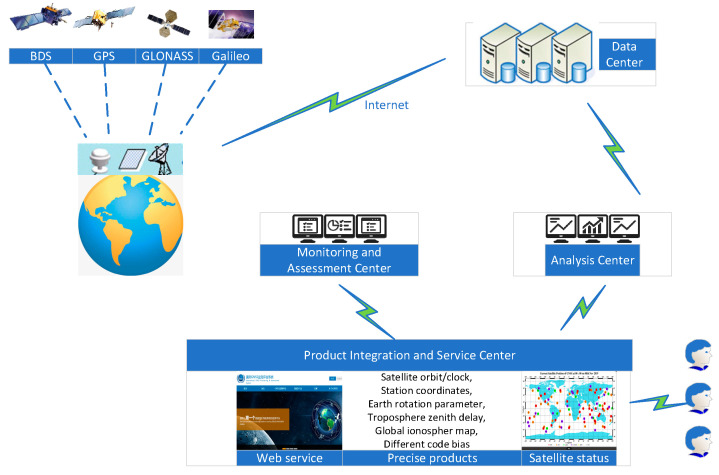
Architecture of iGMAS.

iGMAS is a super-large and complex system, which is composed of several sub-systems including the global tracking stations, the data centers, the analysis centers, the information combination and service center (ISC), the monitoring and assessment center, and the operation control center. Each sub-system supports and connects with each other in function to ensure the stable operation of iGMAS. The iGMAS structure is depicted in Figure 1.

The global tracking stations of about thirty collect multi-GNSS observations and transmit them to the data centers for classification, management, and storage. The analysis centers process the observations collected by tracking stations to generate high-precision GNSS products and submit the products to ISC. Currently, twelve ACs contribute products to iGMAS ISC routinely: Beijing Aerospace Control Center (BAC) [24], Chinese Academy of Surveying and mapping (CGS), Chang’an University (CHD) [25], China University of Mining and Technology (CUM) [26], Institute of Geodesy and Geophysics (CGS), Information Engineering University (LSN) [27], National Time Service Center (NTS) [28], Shanghai Astronomical Observatory (SHA), Tongji University (TJU), Xi’an Research Institute of Surveying and Mapping (XRS) [27], Xi’an Satellite Control Center (XSC) [29], and Wuhan University (WHU) [30].

The ISC is the center of iGMAS products reprocessing, which evaluates the quality of GNSS products submitted by individual analysis centers, and then reprocesses them to generate the combined products and the corresponding accuracy indexes. The products are delivered to users through the portal website of http://www.igmas.org (accessed on 30 December 2021 for Chinese version), which is supported by monitoring stations, data centers, and analysis centers.

The combined products mainly consist of the precise orbits and clocks, the coordinates of tracking stations, the Earth rotation parameters, and atmospheric environment parameters. These combined products are stored and published by data centers, together with the inter-frequency biases, the ionospheric scintillation indexes, and the integrity products provided by the monitoring and assessment center.

Both iGMAS and IGS provide high-precision GNSS products to global users. Although iGMAS and IGS provide the same types of products, iGMAS has achieved, for the first time, the quad-constellation, i.e., BDS/GPS/GLONASS/Galileo combination of the orbit and clock products, whereas IGS only provides GPS/GLONASS combined orbits and GPS-only combined clocks. Until now, the IGS multi-GNSS product combination experiments are still undergoing, and the experimental multi-GNSS combined orbits are available. Please be noted that the time used in iGMAS products is BDT.

## 3. Combination Strategy of Multi-GNSS Orbit and Clock

**Figure 2:**
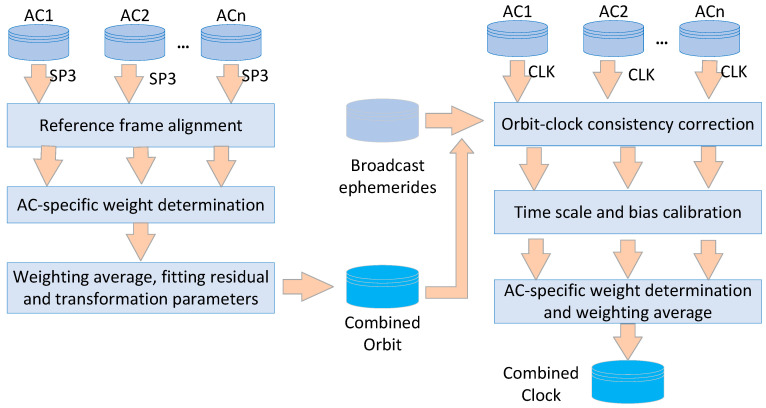
The flowchart of iGMAS orbit and clock combination.

Figure 2 presents the flowchart of multi-GNSS orbit and clock combination of iGMAS. Since both the ACs’ orbits and the combined solutions are used for consistency correction in the procedure of clock combination, the multi-GNSS orbits are firstly combined, then it is the clock combination’s turn. Apart from the orbit and clock products used as input data, the earth orientation parameters and multi-GNSS broadcast ephemerides are also utilized in the combination procedure.

### 3.1. Orbit Combination

Due to different tracking observations and strategies of precise orbit determination adopted by analysis centers, there are systematic biases existing in the terrestrial reference frames (TRF) realized by precise orbits from analysis centers. Therefore, it is necessary to re-process the different orbit solutions to generate combined results, which is consistent with the international TRF (ITRF). Moreover, the combined solutions are more continuous over a large time scale, compared with the lack of some solutions from analysis centers on some days.

Generally, the multi-GNSS orbit combination consists of three steps: the orbital realized TRF calibration, the weight determination for analysis centers, and the orbit averaging.

Step (1): orbital realized TRF calibration. According to [18], the orientation of orbits, station coordinates, and earth rotation parameters (ERP) are highly correlated, it is possible to unify the orientations of the three kinds of products. Since the earth rotation parameters and orbits are simultaneously generated by iGMAS analysis centers, we decide to align the orientation of different orbits to ITRF through ERP, and the following formulas are used.

(1) $$\left\{ \begin{array}{l} {\mathrm{dO}}_{X,AC}^{isat}=-dX_{p}O_{Z,AC}^{isat}\\ {\mathrm{dO}}_{Y,AC}^{isat}=dY_{p}O_{Z,AC}^{isat}\\ {\mathrm{dO}}_{Z,AC}^{isat}=dX_{p}O_{X,AC}^{isat}-dY_{p}O_{Y,AC}^{isat} \end{array}\right.$$

where $dX_{p}$ and $dY_{p}$ are the differences between ERPs of analysis centers and IERS standard EOP data. Due to the inconsistent model used by some analysis centers and insufficient accuracy of orbits for sometimes, the ERP residuals are checked before orientation correction. A threshold of 0.3 mas proposed by IGS is used to detect outlier analysis centers. The analysis centers with ERP residuals larger than 0.3 mas are excluded from the combination.

Step (2): weight determination. The weight of the analysis center is mainly divided into two parts, that is the initial weight and the final weight, and both are calculated by using the root mean square error (RMS) of the analysis center orbit with regard to the combined solutions. The initial combined solutions are derived from the median of all analysis centers for individuals, and the initial weights are identical for all analysis centers. Considering the differences in the accuracy of various satellite orbits, however, we compute weights depending not only on the ACs and satellites but also on the constellations, instead of one weight. Based on the orbits of combined and analysis centers, the observation equation is established as follows, and weights are iteratively updated:

(2) $$O_{AC}^{sys}+v_{AC}^{sys}=O_{cmb}^{sys}+D_{AC}^{sys}+S_{AC}^{sys}+R_{AC}O_{cmb}^{sys}$$

where $O_{cmb}^{sys}$ and $O_{AC}^{sys}$ are the satellite orbits of combined and individual analysis centers for system *sys* (i.e., GPS, GLONASS, BDS, Galileo). $D_{AC}$, $S_{AC}$, and $R_{AC}$ are the translation vector, scale, and rotation matrix, respectively, which can be expressed by $D_{AC}=\left[\begin{array}{c} D_{X,AC}\\ D_{Y,AC}\\ D_{Z,AC} \end{array}\right]$ and $R_{AC}=\left[\begin{array}{ccc} 0 & -\theta_{Z} & \theta_{Y}\\ \theta_{Z} & 0 & -\theta_{X}\\ -\theta_{Y} & \theta_{X} & 0 \end{array}\right]$. $\theta_{X}$, $\theta_{Y}$, and $\theta_{Z}$ are the three rotation angles.

There are some deficiencies in the solar radiation pressure models adopted by analysis centers, especially for the newly constructed global navigation system (e.g., the ECOM-5 without a priori model is used for BDS and Galileo by most analysis centers). Moreover, the PCO and PCV adopted in data processing are not consistent among analysis centers, the values derived either IGS14.atx or calibration of analysis center are used by different analysis centers. As a result, different sets of similarity transformation parameters are designed for each satellite system in this study. Due to the much lower precision of GEO orbits, they are not included in the weight determination. The weights of analysis centers could be calculated based on the RMS derived from orbit residuals as follows:

(3) PACsys=1RMSACsys∑i=1nac1RMSisys

where *nac* indicates the number of analysis centers. $RMS_{AC}^{sys}$ is the RMS of system sys, which can be computed by

(4) $$RMS_{AC}^{sys}=\sqrt{\frac{{{(v}_{AC,cor}^{sys})}^{T}P_{AC}^{sys,Last}v_{AC,cor}^{sys}}{3\cdot np_{AC}^{sys}-7}}$$

In Equation (4), $np_{AC}^{sys}$ is the total epochs of all satellites for system sys, and $P_{AC}^{sys,Last}$ indicates the weight from the last iteration with the initial value of one. Although the least square estimation method adopted by orbit combination is unbiased, it is easy to be affected by the abnormal satellites, which is always the case for the eclipse satellites. As a result, it is necessary to detect and reduce the effects induced by these satellites on the combined solutions. In this paper, the equivalent weight method is used to weaken the impacts of anomalous satellites using the following formula:

(5) $$P_{AC}^{sys,isat}=\left\{ \begin{array}{l} 1,\;\;\;RMS_{AC}^{sys,isat}<3\cdot RMS_{AC}^{sys,med}\\ {\bar{P}}_{AC}^{sys,isat},\;3\cdot RMS_{AC}^{sys,med}\leq RMS_{AC}^{sys,isat}<5\cdot RMS_{AC}^{sys,med}\\ 0,\;\;\;RMS_{AC}^{sys,isat}\geq 5\cdot RMS_{AC}^{sys,med} \end{array}\right.$$

where $RMS_{AC}^{sys,med}$ stands for the median RMS of all satellites for system *sys* and ${\bar{P}}_{AC}^{sys,isat}=2.5-0.5\cdot \frac{RMS_{AC}^{sys,isat}}{RMS_{AC}^{sys,med}}$.

Step (3): orbits averaging. The weighted averaging orbit could be obtained from the multiple ACs using the estimates of transformation parameters and AC specific weight as follows

(6) $$O_{cmb}^{sys,isat}=\frac{\sum_{i=1}^{nac}P_{i}^{sys}{\{O}_{AC,cor}^{sys,isat}-(D_{i}^{sys}+S_{i}^{sys}+R_{i}O_{cor}^{sys,isat})\}}{\sum_{i=1}^{nac}P_{i}^{sys}}$$

It is noticed that the orbits of some satellites are not provided by some of ACs, especially for the satellites during eclipse or attitude switch season. The number of ACs may be different for the two satellites, *isat* and *jsat*, resulting in different weights of the two satellites for the individual ACs. Fortunately, the orbit dynamics are still satisfied approximately when the sum weight of all analysis centers included in the combination for each satellite is one [10].

### 3.2. Clock Combination

In order to keep the consistency between combined clock and orbit solutions, a consistency correction should be applied for clock products of analysis centers by using their corresponding orbits. Meanwhile, the reference clocks and observations adopted by analysis centers are not identical, resulting in satellite-dependent bias between two analysis centers, apart from time scale difference [18,19]. Moreover, differing from the orbits, the clock products are highly susceptible to unoptimized constraints, reference clock, and other unmodelled errors. Generally, the multi-GNSS clock combination can be obtained by the following steps.

Step (1): Consistency correction. To keep the combined clock consistent with the orbit, the orbit-clock consistency correction should be added to the clock products of analysis centers. The correction of the consistency between clock and orbit (i.e., $dC_{AC}^{sys,isat}$) can be calculated using the formula below. Considering that the interval of the orbits are always 900 s, and the sampling intervals of clock products are generally 300 s or less (e.g., 30 s for iGMAS), the Everette interpolation method is adopted in this study to interpolate the orbits [31].

(7) $$\left\{ \begin{array}{l} dC_{AC}^{sys,isat}=e\cdot (O_{AC}^{sys,isat}-O_{cmb}^{sys,isat})\\ e=\left[\begin{array}{c} e_{X}\\ e_{Y}\\ e_{Z} \end{array}\right],e_{X}=\frac{O_{X,cmb}^{sys,isat}}{\rho_{0}},e_{Y}=\frac{O_{Y,cmb}^{sys,isat}}{\rho_{0}},e_{Z}=\frac{O_{Z,cmb}^{sys,isat}}{\rho_{0}} \end{array}\right.$$

where $O_{AC}^{sys,isat}$ and $O_{cmb}^{sys,isat}$ are the orbits of analysis centers and combined one, respectively. e is the unit vector of satellite position.

Step (2): Preprocessing of clock products. Clock preprocessing mainly includes outliers and clock jump detection. First, the clock data are converted into frequency data, and then the abnormal data in the frequency domain are detected by using the robust median detection method. It is easy to find that one frequency outlier indicates the existence of clock jump, and two consecutive outliers in the clock frequency sequence indicate the existence of phase outliers. Second, the corresponding clock phase data for the detected frequency anomaly data are identified separately, and the abnormal value is deleted and the clock phase data are segmented according to the phase jump position.

Step (3): Datum unification for clocks of analysis centers. The GPS clock combination of IGS could be used to align the time scale of clock products of analysis centers to IGST. It is noticed that there is a time delay of few days to two-week delay for the IGS products. As a result, we decided to unify the time scale of clock products to the realization of broadcast clock. Considering the insufficient accuracy of nanoseconds for broadcast clock and using one set of parameters for all satellites cannot completely remove the systematic errors between different AC clocks. It was pointed out that the clock biases among all satellites, caused by realigning the combined GLONASS clock using IGS strategy, make the analysis center unable to obtain a reliable weight, which will eventually affect the reliability of the combined products [20]. Therefore, an approach of linear fitting for individual satellites is used to reduce the satellite-dependent bias and align the clocks to the reference analysis center. Considering their relatively stable performance of precision, we select three analysis centers as the reference clock candidates in this study, that is BAC, SHA, and WHU. Moreover, the analysis center with the best data fitting accuracy and the least phase jump among the three analysis centers is used as the reference. The individual satellite clock is aligned to the reference analysis center by the following formula.

(8) $$C_{ref,t_{k}}^{sys,isat}=C_{AC,t_{k}}^{sys,isat}+a_{o,AC}^{sys,isat}+a_{1,AC}^{sys,isat}\Delta t_{k}$$

where $C_{ref,t_{k}}^{sys,isat}$ and $C_{AC,t_{k}}^{sys,isat}$ are the clocks of reference analysis center and other centers at epoch $t_{k}$, respectively. $a_{o,AC}^{sys,isat}$ and $a_{1,AC}^{sys,isat}$ are the clock offset and drift. $\Delta t_{k}$ is the time span counted from the first epoch and varies from 0 to 24 h.

Step (4): Robust estimation of parameters. The clocks after consistency correction and datum alignment are used to establish the observation equation. The median values are obtained from all analysis centers and taken as the initial combined clock. A similar observation equation as (8) is also established between clocks of individual analysis center and the combined one, and one set of offset and drift for each system is estimated. In order to reduce the impacts of undetected outliers or clock data for satellites during the eclipse, the IGGIII weight function [32] based on posterior residuals is used for the iterative estimation of parameters. The equivalent weight of the satellite *j* of the analysis center *i* at the epoch *k* can be computed by

(9) $$p_{i,k}^{j}=\left\{ \begin{array}{l} 1,\;\;\left|v_{i,k}^{j}\right|<k_{0}\sigma_{i,0}\sqrt{q_{i,k}^{j}}\\ \frac{k_{0}}{\left|v_{i,k}^{j}\right|}\left(\frac{k_{1}\sigma_{i,0}\sqrt{q_{i,k}^{j}}-\left|v_{i,k}^{j}\right|}{k_{1}-k_{0}}\right),k_{0}\sigma_{i,0}\sqrt{q_{i,k}^{j}}\leq \left|v_{i,k}^{j}\right|<k_{1}\sigma_{i,0}\sqrt{q_{i,k}^{j}}\\ 0,\;\;\left|v_{i,k}^{j}\right|>k_{1}\sigma_{i,0}\sqrt{q_{i,k}^{j}} \end{array}\right.$$

where $k_{0}$ generally has a range between 1.0 and 1.5, and $k_{1}$ is selected in the range of 2.5 to 3.0. This paper selects 1.5 and 3.0, respectively. $q_{i,k}^{j}$ is the covariance of posterior residuals of clock products. $\sigma_{i,0}$ is the posteriori unit weight variance factor, which could be calculated by $\sigma_{i,0}=med(\left|v_{i,k}^{j}\right|)/0.6745$, based on the posteriori residuals of all satellite clocks provided by analysis center *i*.

Step (5): Clock-weighted averaging. Considering that the product accuracy of different systems in the same analysis center may be different, the weights are calculated separately for each system and the weighted average clock combination of individual satellites is calculated by the following formula

(10) $$C_{cmb,k}^{j}=\frac{\sum_{i=1}^{nac}w_{i}{\bar{C}}_{i,k}^{j}}{\sum_{i=1}^{nac}w_{i}}$$

where ${\bar{C}}_{i,k}^{j}$ is the calibrated clock of analysis center with consistency correction and alignment of the time scale. $w_{i}$ is the weight of individual analysis centers, which is determined by the weighted RMS of clock residuals for each system by $w_{i}=\frac{1}{{\left(C_{i,RMS}^{sys}\right)}^{2}}$.

## 4. Assessments of iGMAS Orbits and Clocks

In order to verify the effectiveness of the orbit and clock combination strategies in this paper, the multi-GNSS orbits and clocks from 3 January 2021 to 24 April 2021 submitted by iGMAS ACs are collected and used to generate combined solutions. In this section, the internal precisions of combined solutions are first summarized. Then, the consistency between iGMAS and IGS is assessed. In addition, satellite laser ranging (SLR) normal points from ILRS are used to validate the combined orbits. Observations from five iGMAS stations are also collected to conduct precise point positioning (PPP) using iGMAS combined orbits and clocks.

### 4.1. Internal Precisions of Combined Orbits and Clocks

As an internal validation of combined orbits and clocks, the RMS of the differences between the AC products and corresponding combined products is statistically calculated. These statistical values can reflect the internal precision of the combined orbits and clocks. The calculation formula can be as follows:

(11) $$\left\{ \begin{array}{l} \sigma_{O}=\sqrt{\frac{1}{nac}\sum_{iac=1}^{nac}\frac{{{(v}_{O,iac}^{isat})}^{T}P_{O,iac}^{sys}v_{O,iac}^{isat}}{3\cdot np_{iac}^{isat}-7}}\\ \sigma_{C}=\sqrt{\frac{1}{nac}\sum_{iac=1}^{nac}\frac{{{(v}_{C,iac}^{isat})}^{T}P_{C,iac}^{sys}v_{C,iac}^{isat}}{3\cdot np_{iac}^{isat}-2}} \end{array}\right.$$

**Figure 3:**
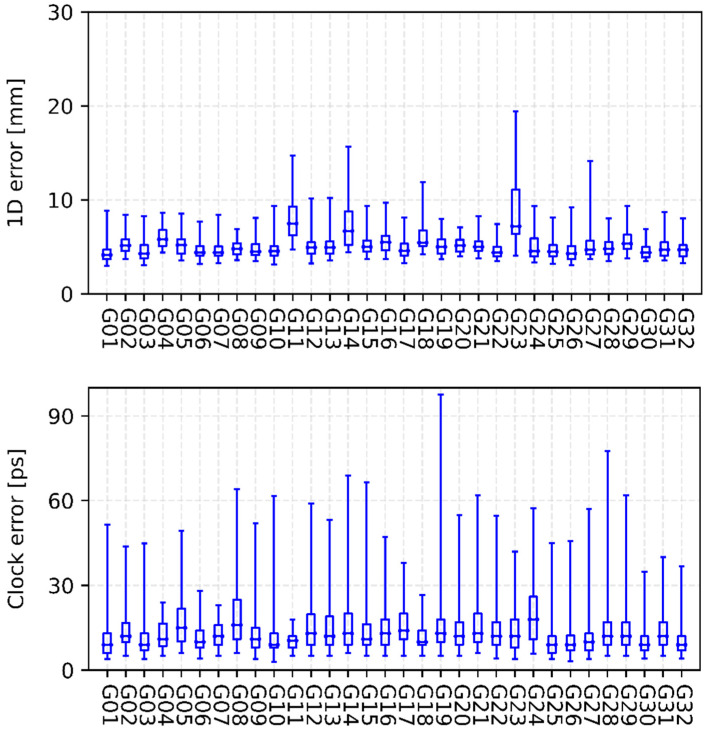
Internal precision of iGMAS final combined GPS orbit (**top panel**) and clock (**bottom panel**).

**Figure 4:**
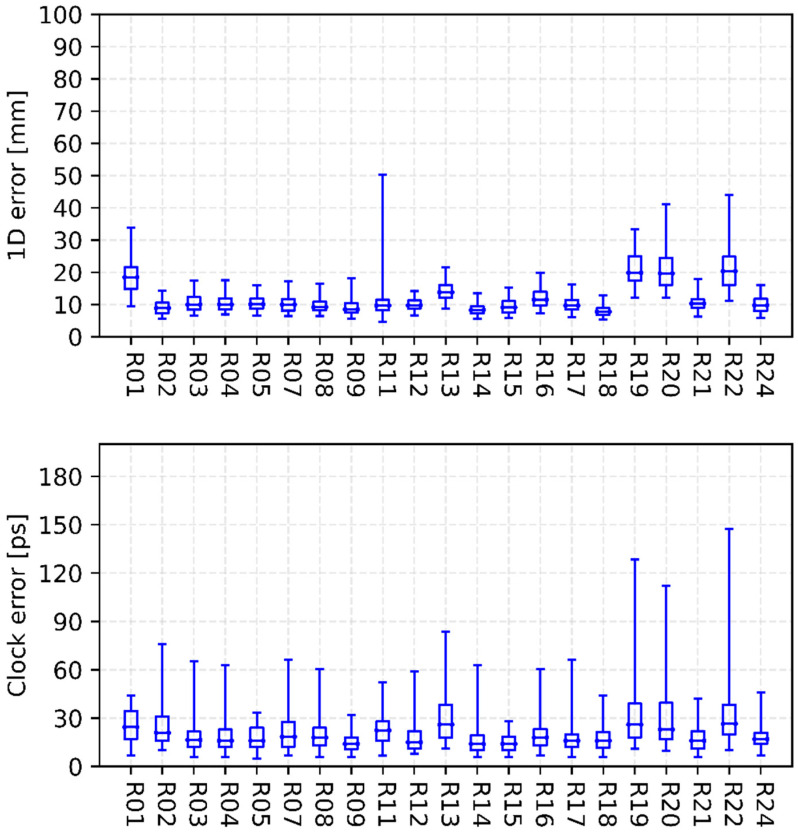
Internal precision of iGMAS final combined GLONASS orbit (**top panel**) and clock (**bottom panel**).

**Figure 5:**
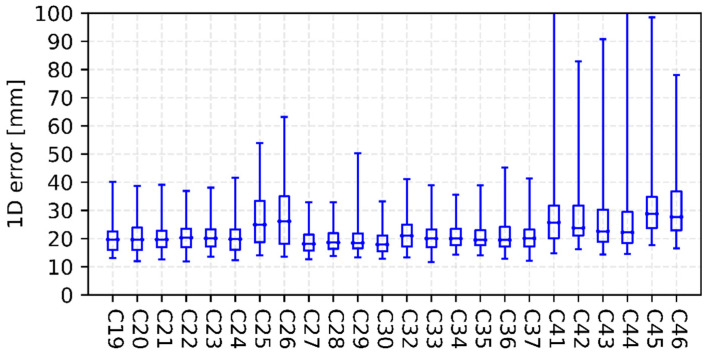

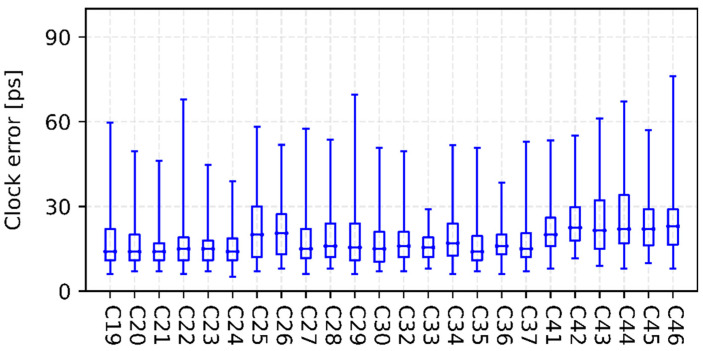
Internal precision of iGMAS final combined BDS-3 MEO orbit (**top panel**) and clock (**bottom panel**).

**Figure 6:**
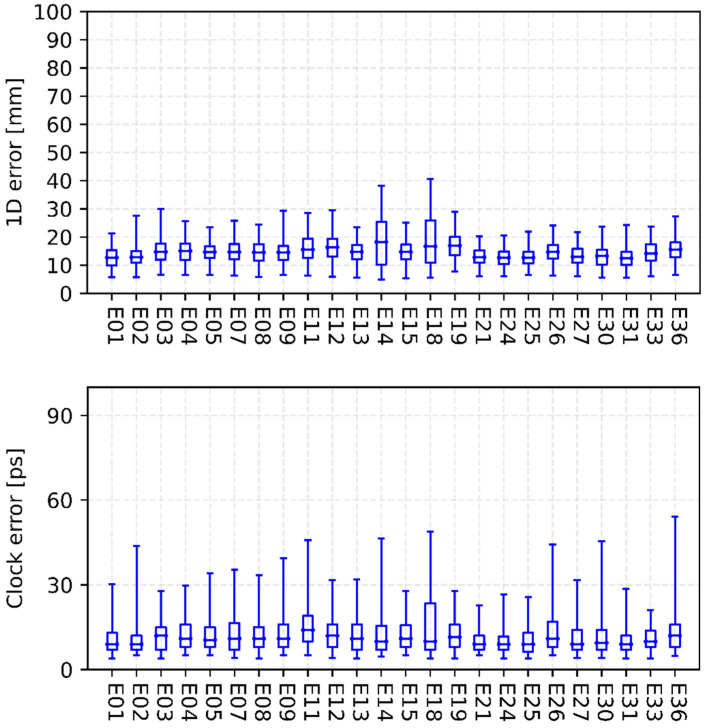
Internal precision of iGMAS final combined Galileo orbit (**top panel**) and clock (**bottom panel**).

Figure 3, Figure 4, Figure 5 and Figure 6 show the internal precision of iGMAS combined GPS, GLONASS, BDS, and Galileo orbits and clocks (for convenient comparison, BDS only gives the results of BDS-3 MEO satellites), respectively. Among the quad constellations, GPS shows the best agreement with respect to combined solutions with an averaged 1D error of 6 mm. It is followed by GLONASS and Galileo, which are at the level of 10–15 mm. The agreement of BDS-3 is the worst at the 20 mm level. In fact, BDS-3 PRN C41-C46 have poorer internal precision due to the relatively small number of global tracking stations. Moreover, the clock errors of GPS, GLONASS, BDS-3, and Galileo are 11, 18, 20, and 15 ps, respectively. Whether for combined orbit or clock, an obvious heavy-tail distribution phenomenon exists in the internal precision, indicating that there are large differences between different AC products. It is worth mentioning that the influence of low-precision products can be partially suppressed by reducing the weight.

### 4.2. Consistency with IGS Orbits and Clocks

**Figure 7:**
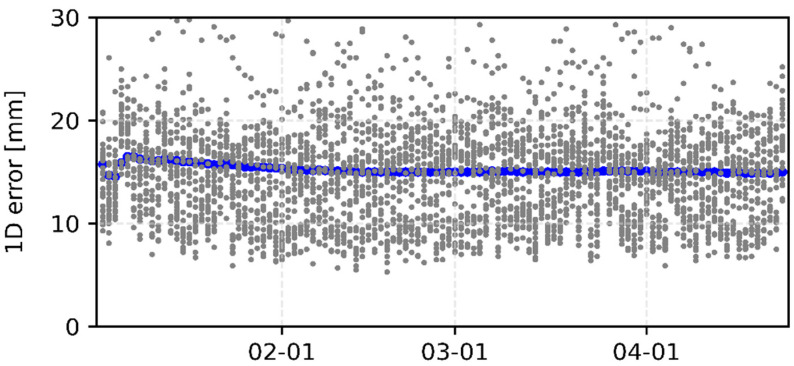
Consistency between iGMAS and IGS final combined GPS orbits. The gray circles indicate each GPS satellite for individual days and the blue circles represent the median RMS of all satellites.

**Figure 8:**
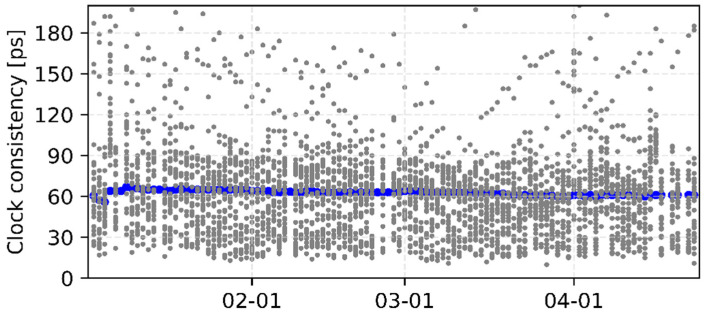
Standard deviations of iGMAS GPS final clocks compared with IGS. The gray circles indicate each GPS satellite for individual day and the blue circles represent the median RMS of all satellites.

**Figure 9:**
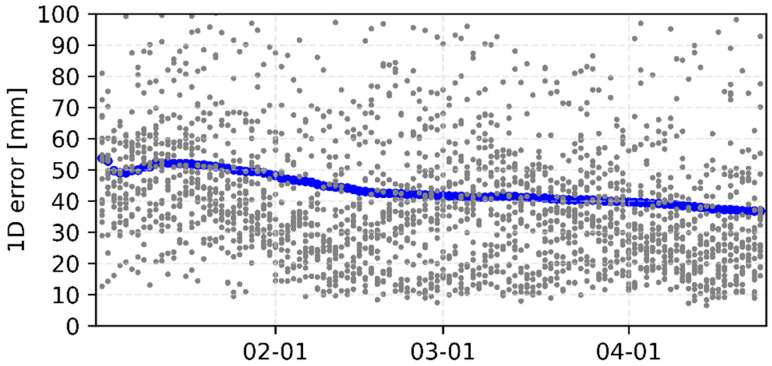
Consistency between iGMAS and IGS final combined GLONASS orbits. The gray circles indicate each GLONASS satellite for individual day and the blue circles represent the median RMS of all satellites.

Figure 7 and Figure 8 show the consistency between iGMAS and IGS final combined GPS orbits and clocks, respectively. The 1D errors of all GPS satellite orbits are basically between 0.6 and 3.0 cm, which are better than 2.0 cm for most satellites. Moreover, the median RMSs of all GPS satellite orbits are relatively stable, with an average value of 1.5 cm. As shown in Figure 7, the standard deviations of clocks mostly show an agreement of 20–90 ps among all GPS satellites, and the median value is 62 ps. In terms of GLONASS, the 1D errors of most satellite orbits are better than 6.0 cm with a median RMS of 4.5 cm (Figure 9).

### 4.3. SLR Validation of iGMAS Combined Orbits

**Figure 10:**
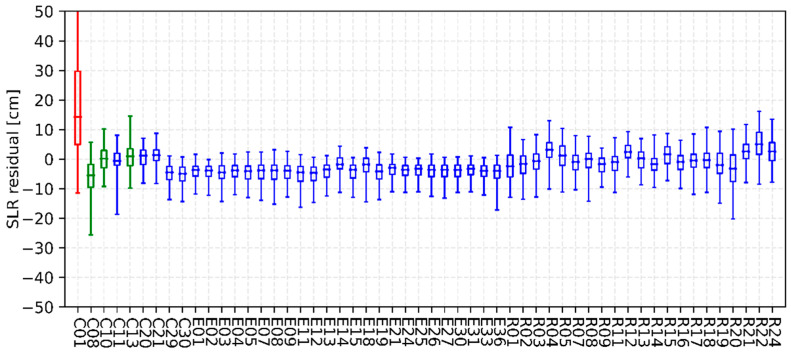
Quantiles of SLR residuals for iGMAS final orbits.

As a key feature, all GLONASS, Galileo and BDS satellites are equipped with laser ranging reflector arrays, enabling high-precision two-way ranging measurements. Thus, satellite laser ranging (SLR) observations collected by the International Laser Ranging Service (ILRS) can be used as an independent validation of iGMAS satellite orbits. Figure 10 shows the quantiles of the SLR residuals for iGMAS final orbits. Table 1 summarizes the mean biases as well as the standard deviations of SLR residuals for iGMAS final orbits. For BDS-2 satellites, the standard deviations are between 4 and 6 cm for MEO and IGSO satellites. However, the SLR residuals show a bias of about 13 cm with a standard deviation of 17.5 cm for C01, which is a GEO satellite. For BDS-3, the orbits of CAST satellites (e.g., C20 and C21) have a bias of 1.2 cm, whereas the orbits of SECM satellites (e.g., C29 and C30) have a bias of −4.7 cm. Moreover, the dispersion degree of SLR residuals for BDS-3 orbits is only 3.5 cm, which is smaller than that of BDS-2. The SLR residuals of Galileo satellites generally have a negative sign of 4.0 cm, which can be explained by the insufficient solar radiation pressure (SRP) modeling, the neglection of the Earth albedo as well as the ignoring of antenna thrust of the Galileo satellites. At the same time, standard deviations of SLR residuals for Galileo and BDS-3 orbits are on a similar level of 3.3 cm. For GLONASS orbits, there is no consistent sign in the bias of SLR residuals, and a standard deviation of 4.2 cm is achieved.

**Figure 11:**
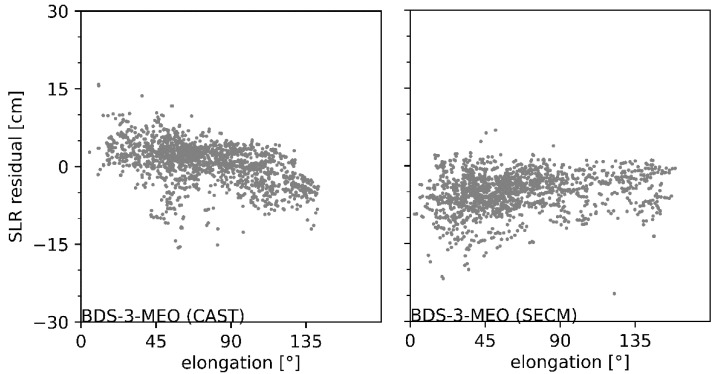
SLR residuals of BDS satellites manufactured by CAST (**left panel**) and SECM (**right panel**) as a function of satellite elongation angle for.

**Figure 12:**
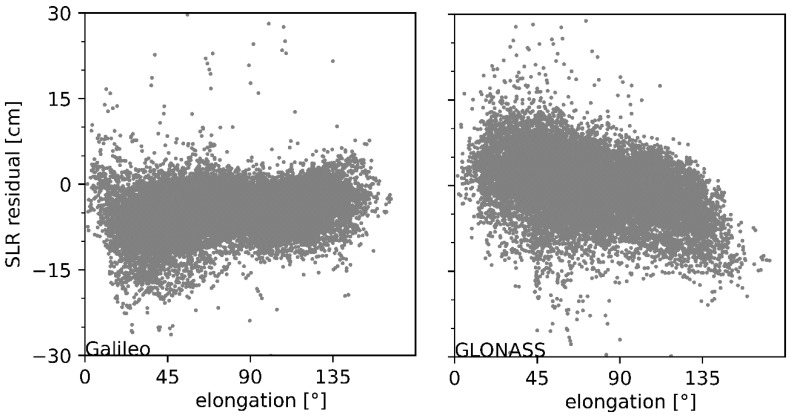
SLR residuals of Galileo (**left panel**) and GLONASS (**right panel**) orbits as a function of satellite elongation angle.

In order to analyze whether a systematic error exists in iGMAS combined orbits, the SLR residuals are shown in Figure 11 as a function of the satellite elongation angle for BDS satellites manufactured by CAST and SECM. It can be seen that the SLR residuals of BDS-3 CAST and SECM satellites show an opposite trend. With the increase in the satellite elongation angle, the SLR residuals of CAST satellites are clearly reduced, whereas the SECM satellites present opposite variations. It is worth mentioning that the variation trend in SLR residuals of Galileo and GLONASS satellites can verify the existence of systematic errors, although the trends are opposite (Figure 12). The systematic errors characterized with a linear pattern in SLR residuals were also validated by [17], which should be attributed to the defective SRP model adopted in precise orbit determination. Therefore, a refined SRP model should be proposed for iGMAS analysis centers [33,34,35,36]. Moreover, the ambiguity-fixed solutions are also encouraged to improve combined solutions for the newly constructed constellation [37].

### 4.4. PPP Using iGMAS Combined Orbits and Clocks

In order to further verify the performance of iGMAS combined orbit and clock products, eight globally distributed stations were adopted for PPP test. The processing strategies employed in PPP are given in Table 2.

**Figure 13:**
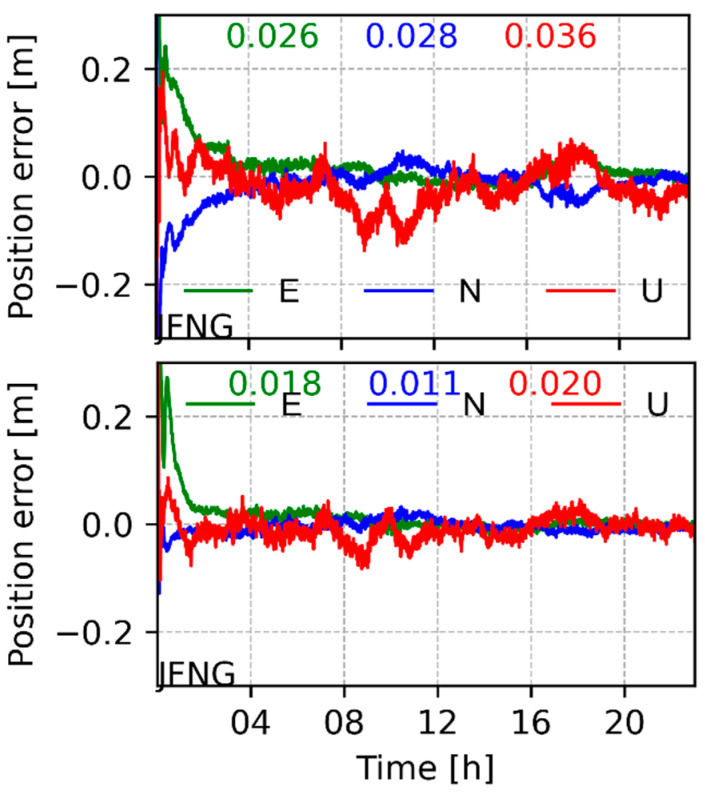
Positioning error of station JFNG in kinematic mode for GPS (**top panel**) and GPS/GLONASS/BDS/Galileo integrated (**bottom panel**) PPP.

Figure 13 shows the kinematic PPP results of GPS-only and GPS/GLONASS/BDS/Galileo multi-system modes at JFNG station on April 24, the last day of the test period. Table 3 summarizes the RMS of each station’s positioning errors. In statistics, the positioning results only show the accuracy after a convergence time of about 1 h (i.e., 120 epochs). It can be clearly seen that the combined GPS/BDS/GLONASS/Galileo solutions significantly shorten the convergence time and improve the position series compared with GPS-only PPP, as shown in the positioning results of the 08:00~12:00 period in Figure 13. For the positioning results of the test station, the average accuracies of GPS-only kinematic PPP are 3.2, 2.2, and 4.9 cm in east, north, and up components, respectively, and those of multi-GNSS kinematic PPP are 1.4, 1.2, and 2.9 cm in east, north, and up components, respectively. Obviously, the multi-GNSS combination significantly improves the PPP performance compared with GPS-only solutions, with improvement rates of 57%, 45%, and 41%, respectively.

## 5. Conclusions

This paper systematically introduces the strategies of orbit and clock combination using multi-GNSS products from iGMAS ACs. In addition, the performance of the combined orbits and clocks is assessed based on half-year products. The results show that the internal precision of 5 mm is achievable for the combined orbits of GPS, whereas the internal precisions of Galileo and BDS are relatively poor at the 1.5 cm level. From the results of SLR validation, there are obvious systematic errors in the iGMAS orbits of different satellite systems, and the systematic errors are manufacturer-dependent. Moreover, there is a negative deviation of 3 cm in the Galileo satellite orbits, which may be attributed to the different non-conservative force models used in ACs’ precise orbit determination processing, such as the Earth albedo radiation and the solar radiation pressure model.

Although the internal precision of iGMAS clock products is basically consistent with the orbit, they are more vulnerable to the influence of observation data quality control and processing strategies. Considering the large fluctuation among various satellite systems, it is necessary to further optimize the quality control before clock combination.

The comparison between iGMAS and IGS GPS orbit/clock shows a consistency of 1.5 cm for orbits and 60 ps for clocks. In addition, the performance of iGMAS combined orbit and clock products is further verified by PPP. As the PPP results show, the average accuracies of multi-GNSS kinematic PPP are 1.4, 1.2, and 2.9 cm in E, N, and U components, respectively; the presenting improvement rates are 57%, 45%, and 41% when compared against the GPS-only PPP solution. In the future, the iGMAS multi-GNSS combined orbit and clock products will be used as reference products for GNSS service performance monitoring and evaluation, and further validation for GNSS geoscience using iGMAS combined solutions is needed.

## Figures and Tables

**Table 1:** Statistics of SLR residuals for iGMAS final combined orbits (unit: cm).

PRN	Bias ± STD	PRN	Bias ± STD	PRN	Bias ± STD
C01	13.0 ± 17.5	E12	−4.5 ± 3.3	R04	3.1 ± 4.9
C08	−5.2 ± 5.8	E13	−3.4 ± 3.2	R05	1.2 ± 5.1
C10	0.1 ± 4.6	E14	−1.7 ± 2.7	R07	−0.9 ± 3.9
C11	−0.5 ± 4.5	E15	−3.5 ± 3.3	R08	0.0 ± 4.6
C13	0.9 ± 5.2	E18	−1.7 ± 3.9	R09	−1.6 ± 3.3
C20	1.1 ± 3.8	E19	−4.1 ± 3.8	R11	−0.9 ± 4.5
C21	1.3 ± 3.2	E21	−2.9 ± 2.8	R12	2.3 ± 3.4
C29	−4.4 ± 3.4	E24	−3.6 ± 2.7	R13	0.1 ± 4.0
C30	−4.9 ± 3.5	E25	−3.2 ± 2.4	R14	−1.7 ± 3.2
E01	−3.6 ± 3.0	E26	−3.6 ± 2.9	R15	1.6 ± 3.9
E02	−3.8 ± 2.5	E27	−3.5 ± 2.8	R16	−0.9 ± 3.6
E03	−4.4 ± 3.3	E30	−3.6 ± 2.9	R17	−0.4 ± 4.1
E04	−3.7 ± 3.1	E31	−3.3 ± 2.8	R18	−0.3 ± 4.7
E05	−4.1 ± 3.7	E33	−3.9 ± 2.6	R19	−2.0 ± 5.4
E07	−3.7 ± 3.5	E36	−3.9 ± 3.4	R20	−3.1 ± 6.6
E08	−3.8 ± 3.7	R01	−2.4 ± 5.2	R21	2.5 ± 4.3
E09	−3.9 ± 3.5	R02	−1.6 ± 4.7	R22	5.0 ± 5.8
E11	−4.5 ± 3.9	R03	−0.6 ± 4.6	R24	2.5 ± 5.1

**Table 2:** Processing strategies applied for PPP tests.

GNSS Considered	GPS, GLONASS, Galileo and BDS
Processing mode	Kinematic
Observables	Undifferenced ionosphere-free linear combination of dual frequency code and phase observation
Satellite orbit and clock	Final precise products of iGMAS combined orbits and clocks
Elevation mask	10°; elevation-dependent weighting of observations
Tropospheric delay	Dry delay modeled by Saastamoinen Wet delay estimated by white noise
Mapping function	Global Mapping Function [38]
Phase wind-up	Corrected [39]
Site displacements effects	Solid Earth tides and ocean loading are corrected [40]
Sampling	30 s
Estimated parameters	Receiver position, receiver clock bias by white noise, tropospheric wet delay, phase float ambiguity and inter system bias parameters

**Table 3:** Statistics of PPP accuracy using iGMAS combined orbit and clock, unit: cm.

SITE	GPS			GPS + GLONASS + BDS + Galileo		
	E	N	U	E	N	U
ABPO	4.4	1.6	4.4	1.0	0.6	2.6
BRST	4.6	3.5	8.3	1.4	1.4	3.0
CHPI	2.8	1.3	4.6	1.1	0.7	2.9
CUT0	2.8	2.1	4.5	1.7	1.3	2.2
JFNG	2.6	2.8	3.6	1.8	1.1	2.0
KOKB	1.4	1.1	4.3	0.9	0.7	3.1
SAVO	4.9	3.5	7.1	2.7	3.0	5.6
UNB3	1.7	1.6	2.2	0.8	0.8	1.6
Mean	3.2	2.2	4.9	1.4	1.2	2.9

## Data Availability

The precise orbit and clock products of iGMAS analysis centers, as well as the combined solutions conducted by this study can be made available upon request by contacting the corresponding author, G.C. by email.
